# Knowledge, attitudes and practices related to SARS-CoV-2 prevention in Kenya

**DOI:** 10.4102/jphia.v16i1.1401

**Published:** 2025-10-23

**Authors:** Brennan R. Cebula, Roger Ying, Tyler Hamby, Julius Tonzel, Josphat Kosgei, Deborah Langat, Rael Bor, Britt Gayle, Matthew L. Romo, Glenna Schluck, Christine Akoth, Fred Sawe, Margaret Yacovone, Julie A. Ake, Trevor A. Crowell

**Affiliations:** 1 US Military HIV Research Program, Center for Infectious Disease Research, Walter Reed Army Institute of Research, Silver Spring, United States of America; 2 Department of Global Infectious Diseases, Henry M. Jackson Foundation for the Advancement of Military Medicine, Bethesda, United States of America; 3 HJF Medical Research International, Kericho, Kenya; 4 US Army Medical Research Directorate – Africa, Kericho, Kenya; 5 National Institute of Allergy and Infectious Diseases, National Institutes of Health, Rockville, United States of America

**Keywords:** HIV, SARS-CoV-2, COVID-19, non-pharmaceutical intervention, knowledge, attitudes and practices

## Abstract

**Background:**

Knowledge, attitudes, and practices (KAPs) regarding severe acute respiratory syndrome coronavirus 2 (SARS-CoV-2) non-pharmaceutical interventions (NPIs) may differ among populations with health vulnerabilities.

**Aim:**

To examine COVID-19 KAPs among Kenyan adolescents and adults with behavioural vulnerability to HIV.

**Setting:**

This study was conducted in Kericho and Homa Bay, Kenya.

**Methods:**

From December 2021 to April 2023, we enrolled participants without HIV aged 14-55 years who reported recent sexually transmitted infection, injection drug use, transactional sex, condomless sex, and/or anal sex with males. A self-administered questionnaire captured sociodemographic data and KAPs. Multivariable robust Poisson regression with purposeful variable selection was used to estimate prevalence ratios (PRs) and 95% confidence intervals (CIs) for factors associated with NPI practices.

**Results:**

Among 399 participants (median age 22 years [interquartile range 19–24]), 317 (79.4%) were female. Participants during the Omicron-variant wave were less likely to meet outdoors (PR = 0.85 [95% CI: 0.73–0.98]), reduce shopping (PR = 0.83 [95% CI: 0.73–0.96], and avoid crowds (PR = 0.81 [95% CI: 0.71-0.93]). Believing that mask-wearing prevents SARS-CoV-2 was associated with less meeting outdoors (PR = 0.44 [95% CI: 0.27–0.73]) and reducing shopping (PR = 0.48 [95% CI: 0.31–0.76]), while believing that handwashing prevents SARS-CoV-2 was associated with less crowd avoidance (PR = 0.73 [95% CI: 0.60–0.89]). Perceiving widespread community face mask use was associated with reduced shopping (PR = 1.12 [95% CI: 1.02–1.23]).

**Conclusion:**

Belief in personal NPIs (mask-wearing and handwashing) was associated with decreased practice of social NPIs (meeting outdoors, reducing shopping, and avoiding crowds).

**Contribution:**

Future public health strategies for pandemic response should anticipate risk compensation.

## Introduction

Early in the COVID-19 pandemic, non-pharmaceutical interventions (NPIs) such as face masks, social distancing and hand hygiene were critical to decreasing severe acute respiratory syndrome coronavirus 2 (SARS-CoV-2) transmission.^1,2^ Governments often relied on mandates to encourage individual adherence to NPIs to control the pandemic. Overall, NPIs were effective at reducing viral transmission, but there was notable heterogeneity in effectiveness around the world because of both country and individual-level factors.^3^

### The COVID-19 non-pharmaceutical interventions background

To inform efforts addressing individual-level factors relating to NPI effectiveness, research has focused on understanding knowledge, attitudes and practices (KAPs) regarding COVID-19. These efforts were particularly important in many African countries where delayed access to vaccines resulted in increased reliance on NPIs.^4,5,6^ Knowledge gained from this sort of research has become critical to both addressing the COVID-19 pandemic and preparing for future similar pandemics.

### Knowledge, attitudes and practice research in the world and Africa

A review of 84 studies across 45 countries found wide variations in KAPs, particularly for adherence to accepted COVID-19 NPIs, which ranged from 27% to 97% with the lowest levels of adherence in low- and middle-income European and African countries.^7^ Furthermore, although knowledge regarding COVID-19 might be expected to translate to NPI practice, this has not been consistently true. A review of studies in healthcare workers around the world found adherence to accepted practices to vary between 30% and 90% despite knowledge of NPIs.^8^ A scoping review of COVID-19 KAPs in sub-Saharan Africa similarly found many studies demonstrating discrepancies between disease knowledge and use of NPIs.^9^ Finally, patterns of COVID-19 KAPs have also varied, with a study in Ethiopia noting a positive correlation between knowledge and practice,^10^ whereas a study in Kenya found a negative correlation between knowledge and practice.^11^ The Health Belief Model, which suggests that individuals weigh perceived benefits of protective behaviours against perceived barriers to implementation, provides a framework for understanding how perceived susceptibility, severity, benefits and barriers influence health behaviours such as NPI adoption. Taken together, these studies highlight the complexity among KAPs for COVID-19 in countries vulnerable to future pandemics.

### Vulnerable populations and HIV and COVID-19 overlap

For individuals with high risk of COVID-19 and its sequelae, understanding KAP patterns is crucial. Populations with behavioural vulnerability to human immunodeficiency virus (HIV), such as individuals who engage in sex for income or inject drugs, may also have particular risk for COVID-19, given overlapping social determinants for COVID-19 and HIV.^12,13^ A study within the United States (US) examining the incidence of COVID-19 and HIV found a spatial correlation between the two.^13^ Factors that contributed to the correlation through associations with both COVID-19 and HIV included binge drinking, chlamydia and income inequality.^13^ Finally, social stigma towards individuals with behavioural vulnerability to HIV has been found to increase perceived healthcare stigma, potentially creating barriers to healthcare.^14,15,16,17,18^ In Kenya, the first cases of SARS-CoV-2 infection were diagnosed in March 2020, although the virus was likely circulating even earlier, with lockdowns and other nationwide mitigation efforts implemented almost immediately thereafter.^19^ Two studies identified large barriers to providing HIV testing because of social stigma that were exacerbated by COVID-19 policies such as restrictions on clinic capacity and operating hours.^20,21^ Transient reductions in HIV viral load testing and food security were observed early in the pandemic in several African countries.^22^ Therefore, understanding COVID-19 KAPs in populations with high HIV incidence is critical to preventing further COVID-19-related morbidity and mortality.

### HIV and COVID-19 in Kenya

Kenya has a particularly large burden of HIV with an estimated national adult HIV prevalence in 2023 of 3.2%.^23^ There is geographic heterogeneity in Kenya’s HIV epidemic with HIV prevalence of over 16% in certain regions.^24^ The Kenyan HIV epidemic also disproportionately affects specific subgroups of the general population such as men who have sex with men (MSM); people who engage in transactional sex; and adolescent girls and young women.^25,26^ Understanding COVID-19 KAPs among these populations is crucial given their shared barriers to healthcare access. We used cross-sectional questionnaire data from enrolment into an observational cohort of people with behavioural vulnerability to HIV in Western Kenya to examine COVID-19 KAPs with the goal of informing strategies to decrease transmission risk of SARS-CoV-2 and other future pathogens.

## Research methods and designs

### Study design

This study is a cross-sectional analysis of questionnaire data collected at enrolment into a longitudinal cohort study.

### Setting

Participants were recruited from two sites in Kenya: the Kericho Clinical Research Center in Kericho and its satellite location in Homa Bay. Sites were selected because of a long-standing partnership between the US Military HIV Research Program and the Kericho Clinical Research Center.

### Study population and sampling strategy

From December 2021 to March 2023, the Multinational Observational Cohort of HIV and other Infections (MOCHI) enrolled individuals aged 14–55 years without HIV. Participants were recruited through community outreach from hotspot areas in Kericho and Homa Bay. Participants were invited to the clinic for a briefing session, and if interested in participating in the study, were consented and screened for eligibility. Participants aged as young as 14 years were included because of known early sexual debut in Western Kenya region and previous data showing that sexual behaviours associated with vulnerability to HIV and other sexually transmitted infections (STIs) tend to be common in the years following sexual debut.^27,28,29^ The primary objective of the longitudinal cohort study was to estimate HIV incidence within populations with behavioural vulnerability to HIV. Behavioural vulnerability was defined as satisfying one or more of the following criteria in the 24 weeks prior to screening for study eligibility: (1) newly diagnosed syphilis, gonorrhoea, chlamydia, *Mycoplasma genitalium*, herpes simplex virus and/or acute hepatitis C virus; (2) engagement in vaginal, oral or anal intercourse in exchange for money as a regular source of income; (3) engagement in condomless vaginal or anal intercourse with at least three different partners living with HIV or of unknown status; (4) engagement in injection drug use or (5) engagement in insertive or receptive anal intercourse with one or more different male partners. These characteristics do not represent the general population, but reflect the target population of participants with high risk of STIs. Exclusion criteria included individuals who were already living with HIV, pregnancy at the time of screening, diagnosis of a substance dependence that would impair participation in the study or having previously received an investigational HIV vaccine. These exclusion criteria were included to mimic a study population that may someday be recruited into a clinical trial of an HIV prevention product.

Sample size for the cohort was calculated based on a goal of confidently detecting an HIV incidence rate of at least 3 cases per 100 person-years. With target accrual of 400 participants and an anticipated dropout rate of 30%, 19 incident HIV cases would need to be observed to determine with 95% confidence that the true HIV incidence was no less than 3 cases per 100 person-years (estimated incidence 4.90 [95% CI: 3.01–7.45] per 100 person-years). These cross-sectional analyses included all participants who completed a COVID-19 questionnaire at enrolment.

### Data collection

At enrolment, participants completed questionnaires using computer-assisted self-interview. The socio-behavioural questionnaire ascertained, among other questions, if participants were male and reported male sex partners in the past 12 weeks or engaged in transactional sex (responding ‘Yes’ to ‘Are you a person who is a sex worker [sex in exchange for things such as money, drugs, food, shelter or transportation]?’). The questionnaire also ascertained participant weekly income, which was dichotomised to ≤ KES 1500.00 (Kenyan shillings) and > KES 1500.00, which is approximately the 2023 Kenyan poverty level.^30^

Questionnaire items were designed to assess key constructs from behavioural theory including perceived benefits (knowledge statements), perceived barriers (attitude questions) and behavioural outcomes (practice measures). The COVID-19 questionnaire focused on COVID-19 KAPs in the preceding month, including any use of NPIs to prevent SARS-CoV-2 acquisition or transmission. Coronavirus disease 2019 knowledge was assessed with two questions. The first question asked participants to ‘Please classify the following statements as either “true” or “false”’ with statements regarding SARS-CoV-2 transmission (e.g., ‘COVID-19 can be transmitted through coughing or sneezing’). Responses were dichotomised to true versus false or don’t know. The second question asked participants ‘For each behaviour, please indicate the extent to which you agree or disagree that it prevents the spread of COVID-19’ with behaviours including personal NPIs (e.g., handwashing) and social NPIs (e.g., reducing shopping trips). Responses were dichotomised to agree (strongly agree or somewhat agree) or disagree (strongly disagree, somewhat disagree, neutral or don’t know).

COVID-19 attitudes were assessed by asking participants two questions. The first question ascertained participants’ opinions regarding COVID-19. Participants were asked, ‘Please select the answer depending on how much you agree with each statement below’ with statements such as ‘My becoming infected with COVID-19 poses a risk to others’. Responses were dichotomised to agree (strongly agree or somewhat agree) versus disagree (strongly disagree, somewhat disagree, neutral or don’t know). The second question ascertained participants’ beliefs regarding how their communities behaved. Participants were asked ‘In your community, to what extent do you think that people do the following when they go out in public?’ with statements of social distancing and wearing a face mask. Responses were dichotomised to always or often (always or often) versus occasionally or less (occasionally, rarely, never, or do not know).

COVID-19 prevention practices were assessed by asking participants ‘In the past month, what measures have you taken to prevent infection from COVID-19?’ with a list of personal NPIs (e.g., face masks) and social NPIs (e.g., meeting in the open). Responses were dichotomised into yes or no (no, don’t know, refuse to answer or no response). To assess personal experience with COVID-19, participants were also asked ‘Have you been infected with COVID-19’ and ‘Do you personally know anyone other than yourself who has been told by a doctor that they have COVID-19?’, and responses were dichotomised into yes (Yes confirmed or Yes suspected but not confirmed) or no (No, do not suspect or don’t know).

The date of questionnaire completion was recorded and later assigned to a SARS-CoV-2-variant wave, if applicable. Waves were defined to begin on the first day of two consecutive 7-day periods with > 20% increase in COVID-19 cases.^31^ Waves were defined to end where two consecutive 7-day periods had fewer cases than at the beginning of the wave or at the beginning of two consecutive 7-day periods with < 10% absolute weekly change in the number of cases, whichever occurred first. The waves were defined as Omicron (29 November 2021 – 14 February 2022), Omicron BA.4/BA.5 (02 May 2022 – 22 August 2022) and Omicron BQ1/BQ.1.1 (10 October 2022 – 02 January 2023) (Appendix 1 Figure 1-A1).^32^

### Data analysis

In total, there were 28 potential independent variables including demographic items, COVID-19 knowledge, COVID-19 attitudes, personal experience with COVID-19 and COVID-19 wave. Given that COVID-19 NPIs directly decrease SARS-CoV-2 transmission, NPIs were the outcome variables of interest. NPIs that had near universal agreement were described but excluded from further analyses. Descriptive statistics included median and interquartile range (IQR) for continuous variables, and frequency and percent for categorical variables, which were estimated overall and stratified by site. Missing data for descriptive statistics were addressed by using an available-case approach, which utilised all data available for each descriptive analysis. Robust Poisson regression was used to estimate prevalence ratios (PRs) and 95% confidence intervals (CIs) for all inferential analyses. Univariable analyses were performed to estimate unadjusted PRs and 95% CIs for the relationship between each independent variable and each NPI. In multivariable analyses for each COVID-19 NPI, purposeful variable selection was used to determine which of the 28 candidate independent variables to include in each model.^33^

Purposeful variable selection included the following four steps: (1) Univariable regression analyses were run for each candidate predictor variable. Those variables with *p* < 0.25 were then entered into a multivariable model. (2) One variable at a time was removed among those with *p* > 0.05 in the multivariable model from step 1. If a variable had *p* > 0.05 but its removal resulted in a > 20% change in one or more other regression coefficient from step 1, then that variable was retained; otherwise, that variable was removed. This process continued until all variables with *p* > 0.05 were examined. The remaining variables were retained for all further analyses. (3) Next, each of those variables that had *p* > 0.25 in univariable analyses (step 1) were added, one at a time, to the multivariable model (from step 2). Any variables with *p* < 0.05 were included in the final model. (4) Lastly, diagnostic checks were performed on the final model from step 3. Missing data for regression analyses were addressed by using a complete-case approach, which included only participants with non-missing variables for all candidate variables; this restricted analytic population was used for each univariable regression and the final multivariable regression.

Statistical analyses were performed using SAS version 9.4 software (SAS Institute, Cary North Carolina, US). Figures were developed using R version 4.2.2 (R Foundation for Statistical Computing, Vienna, Austria). *P*-values < 0.05 were considered statistically significant. *P*-values were not corrected for multiple comparisons.

### Ethical considerations

Ethical clearance to conduct this study was obtained from the Walter Reed Army Institute of Research (No. WRAIR #2877B). The investigators have adhered to the policies for protection of human research participants as prescribed in Army Regulation 70-25. All participants provided written informed consent prior to any study procedures. Participants aged 14–17 years with behavioural vulnerability to HIV and other STIs were considered outside of parental influence or control and provided their own informed consent as emancipated minors (mature minors), consistent with the Guidelines for Conducting Adolescent HIV Sexual and Reproductive Health Research in Kenya. Procedures for participant protection and confidentiality included the use of study identification numbers and locked, secured data storage. The study was approved by institutional review boards at the Kenya Medical Research Institute, the Walter Reed Army Institute of Research and all collaborating institutions.

## Results

### Participants’ demographics

Of the 486 participants screened, 413 were deemed eligible, of whom 407 enrolled in the study. Of the 407 participants enrolled in Homa Bay and Kericho, Kenya, 399 (98.0%) completed any of the COVID-19 questionnaire (Table 1). Their median age was 22 years (Q1–Q3 19–24), with 318 (79.7%) participants between 15 years and 24 years old. Key demographic patterns showed predominance of young females (79.4%) engaged in transactional sex (79.2%), with most participants having secondary education or less (65.9%).

**TABLE 1:** Enrolment characteristics of participants with behavioural vulnerability to HIV in Homa Bay and Kericho, Kenya (*N* = 399).

Characteristic	Overall		Kericho (*n* = 181, 45.4%)		Homa Bay (*n* = 218, 54.6%)		*p* *
	*N*	%	*n*	%	*n*	%	
**Age (years)**	-	-	-	-	-	-	< 0.001
14–24	318	79.7	180	99.4	138	63.3	-
25–55	81	20.3	1	0.6	80	36.7	-
**Sex**	-	-	-	-	-	-	< 0.001
Female	317	79.4	165	91.2	152	69.7	-
Male	82	20.6	16	8.8	66	30.3	-
**Men who have sex with men**	-	-	-	-	-	-	0.007
Yes	45	11.3	12	6.6	33	15.1	-
No	351	88.0	168	92.8	183	83.9	-
Missing	3	0.8	1	0.6	2	0.9	-
**Marital status**	-	-	-	-	-	-	< 0.001
Currently married	14	3.5	0	0.0	14	6.4	-
Separated or divorced	49	12.3	16	8.8	33	15.1	-
Single or never married	324	81.2	157	86.7	167	76.6	-
Widowed	1	0.3	1	0.6	0	0.0	-
Missing	11	2.8	7	3.9	4	1.8	-
**Years of education**	-	-	-	-	-	-	< 0.001
≤ 12	263	65.9	146	80.7	117	53.7	-
> 12	135	33.8	34	18.8	101	46.3	-
Missing	1	0.3	1	0.6	0	0.0	-
**Engagement in transactional sex**	-	-	-	-	-	-	< 0.001
Yes	316	79.2	176	97.2	140	64.2	-
No	74	18.5	3	1.7	71	32.6	-
Missing	9	2.3	2	1.1	7	3.2	-
**Weekly income†**	-	-	-	-	-	-	0.232
≤ 1500.00 Kenyan shillings	208	52.1	100	55.2	108	49.5	-
>1500.00 Kenyan shillings	190	47.6	80	44.2	110	50.5	-
Missing	1	0.3	1	0.6	0	0.0	-
**COVID-19 wave**	-	-	-	-	-	-	< 0.001
Non-wave	152	38.1	68	37.6	84	38.5	-
Omicron: 29 November 2021 – 14 February 2022	31	7.8	0	0.0	31	14.2	-
Omicron BA.4/BA.5: 02 May 2022 – 22 August 2022	132	33.1	53	29.3	79	36.2	-
Omicron BQ.1/BQ.1.1: 10 October 2022 – 02 January 2023	84	21.1	60	33.1	24	11.0	-

Note: From December 2021 to March 2023, participants without HIV were recruited in Homa Bay and Kericho, Kenya. Statistically significant comparisons (*p* < 0.05) are shown in bold.

HIV, human immunodeficiency virus.

* , Chi-squared tests were used to compare participant characteristics from the two sites.

† , Dichotomised at approximately the poverty level.

### Knowledge about COVID-19

Knowledge of COVID-19 NPIs was generally high, with over 80% of respondents agreeing that each of the solicited NPIs may reduce the risk of SARS-CoV-2 transmission (Figure 1a). When asked ‘In the past month, what measures have you taken to prevent infection from COVID-19’, the NPIs with the most agreement were face masks (*n* = 353 participants, 88.5%), social distancing (*n* = 347, 87.0%) and handwashing (*n* = 343, 86.0%). The NPIs with the lowest agreement were reducing shopping trips (*n* = 325, 81.5%) and meeting in the open (*n* = 326, 81.7%) (Appendix 1 Table 1-A1).

**FIGURE 1:**
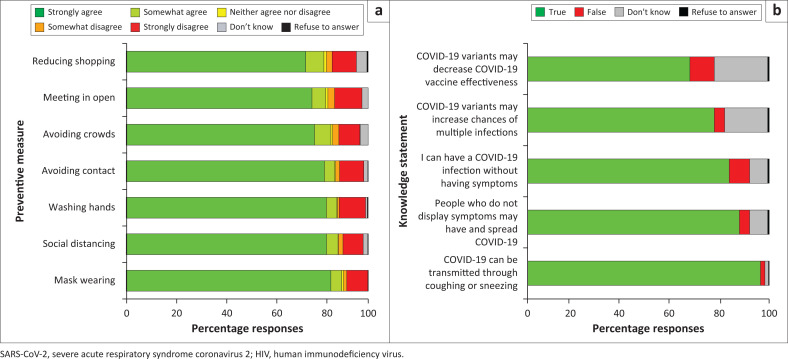
Distribution of responses to questions regarding knowledge of SARS-CoV-2 non-pharmaceutical interventions to prevent COVID-19 (a) and transmission (b) among participants with behavioural vulnerability to HIV in Kenya.

However, COVID-19 knowledge varied (Figure 1b). Nearly all participants correctly identified that transmission can occur through coughing or sneezing (*n* = 384, 96.2%). Over 80% of participants correctly identified that it is possible to have no symptoms from SARS-CoV-2 infection (*n* = 333, 83.5%) and also to transmit the virus without having symptoms (*n* = 350, 87.7%). However, there was lower knowledge of SARS-CoV-2 genetic variants, with smaller majorities of participants correctly identifying that SARS-CoV-2 variants can have differing virulence (*n* = 309, 77.4%) and that vaccines have lower effectiveness in preventing some SARS-CoV-2 variants (*n* = 268, 67.2%) (Appendix 1 Table 2-A1).

### Attitudes about COVID-19

**FIGURE 2:**
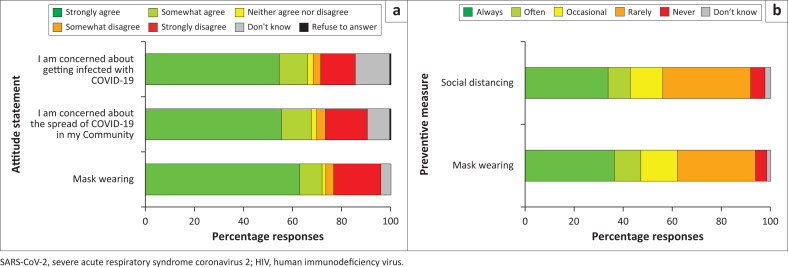
Attitudes regarding COVID-19 risk (a) and perceptions of community practices (b) among participants with behavioural vulnerability to HIV in Kenya.

Attitudes about COVID-19 were assessed for one’s personal risk and belief in their community’s actions. When asked about the risk of SARS-CoV-2 infection (Figure 2a), most participants agreed that becoming infected with COVID-19 poses a risk to others (*n* = 287, 71.9%). Most participants were also concerned about the spread of COVID-19 in their communities (*n* = 270, 67.7%) and getting infected with COVID-19 (*n* = 263, 65.9%) (Appendix 1 Table 3-A1). However, fewer than half of participants believed that people in their community used NPIs such as social distancing (*n* = 171, 42.9%) and wearing face mask (*n* = 188, 47.1%) (Figure 2b, Appendix 1 Table 4-A1).

### Uptake of non-pharmaceutical interventions to prevent COVID-19

**FIGURE 3:**
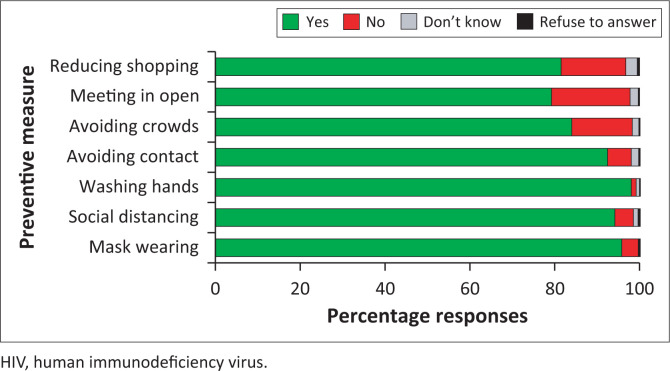
Practice of non-pharmaceutical interventions to prevent COVID-19 among participants with behavioural vulnerability to HIV in Kenya.

Among the COVID-19 NPIs, personal NPIs were the most practiced in the month prior to enrolment including handwashing (*n* = 390, 97.7%) and mask-wearing (*n* = 380, 95.2%) (Figure 3). Furthermore, social distancing and avoiding contact with sick individuals were also endorsed by 372 (93.2%) and 365 (91.5%) respondents, respectively. The least endorsed measures were the social NPIs of meeting in the open (*n* = 312, 78.2%), reducing shopping (*n* = 325, 81.5%) and avoiding crowds (*n* = 331, 83.0%). There were 250 (62.7%) participants who endorsed adhering to all seven NPIs (Appendix 1 Table 5-A1).

### Factors associated with non-pharmaceutical intervention uptake

Among the COVID-19 NPIs, the social NPIs of meeting in the open, reducing shopping and avoiding crowds were modelled as outcome variables given their lower levels of uptake. Among the 399 participants who completed any of the COVID-19 questionnaire, 349 participants completed the entire questionnaire and were included in inferential analyses. In univariable analyses (Appendix 1 Table 6-A1 to Table 8-A1), participants responding during the Omicron BA.4/BA.5 wave were less likely to endorse reducing shopping and avoiding crowds (PR = 0.85 [95% CI: 0.76–0.96] and 0.87 [95% CI: 0.77–0.97], respectively). Knowing someone previously diagnosed with COVID-19 was associated with increased probability of avoiding crowds (PR = 1.10 [95% CI: 1.00–1.20]). Broadly, participants with correct knowledge of COVID-19 (asymptomatic transmission and of the impact of genetic variants on transmission) and of NPIs relating to reducing close contact (reducing shopping trips and meeting in the open) were more likely to endorse practicing those same NPIs. Finally, attitudes regarding individual concern for COVID-19 and community use of NPIs were not related to practice of any of the NPIs evaluated.

In adjusted analyses, purposeful variable selection improved the model performance compared to models that included all variables. For meeting in the open, purposeful variable selection resulted in a model with Akaike information criterion (AIC) of 696.8 compared to AIC of 725.3 for the full model. For reducing shopping, the AIC after purposeful variable selection was 694.1 compared to 726.5 for the full model. For avoiding crowds, the AIC after purposeful variable selection was 714.0 compared to 733.9 for the full model.

Male sex and engagement in transactional sex were positively associated with meeting in the open (adjusted PR [aPR] = 1.27 [95% CI: 1.11–1.45] and 1.24 [95% CI:1.02–1.50], respectively) (Table 2). Older participants (25–55 years vs. 14–24 years) and those who were married were more likely to reduce shopping (aPR = 1.13 [95% CI: 1.00–1.28] and 1.19 [95% CI: 1.01–1.41], respectively) (Table 3). Finally, participants with more years of education (> 12 years vs. < 12 years) were more likely to avoid crowds (aPR = 1.19 [95% CI:1.08–1.31]) (Table 4).

**TABLE 2:** Multivariable analyses of factors associated with endorsement of meeting in the open to prevent COVID-19 (*n* = 349, AIC = 696.8).

Variable	Adjusted prevalence ratio	95% confidence interval
**Demographic variables**		
Sex		
Female	Ref	Ref
Male	1.27	1.11–1.45
Site		
Homa Bay	Ref	Ref
Kericho	1.09	0.95–1.26
Engagement in transactional sex		
No	Ref	Ref
Yes	1.24	1.02–1.50
**COVID-19 variables**		
COVID-19 wave		
Non-wave	Ref	Ref
Omicron: 29 November 2021 – 14 February 2022	0.85	0.73–0.98
Omicron BA.4/BA.5: 02 May 2022 – 22 August 2022	0.94	0.84–1.06
Omicron BQ.1/BQ.1.1: 10 October 2022 – 02 January 2023	1.09	0.87–1.37
**Knowledge statements**		
COVID-19 can be transmitted through coughing or sneezing		
False	Ref	Ref
True	1.33	0.75–2.36
People who do not display symptoms may still be infected with COVID-19		
False	Ref	Ref
True	1.08	0.86–1.36
Variants (mutated strains) of COVID-19 may increase the chance that people can be infected with COVID-19		
False	Ref	Ref
True	1.05	0.89–1.23
Avoiding close contact with anyone who has fever and/or cough		
Disagree	Ref	Ref
Agree	1.27	0.82–1.98
Wearing a face mask or covering correctly		
Disagree	Ref	Ref
Agree	0.44	0.27–0.73
Staying at least 1 metre away from other people when out in public		
Disagree	Ref	Ref
Agree	1.26	0.75–2.12
Meeting others in open spaces rather than indoors		
Disagree	Ref	Ref
Agree	1.57	1.11–2.21
**Attitude statements**		
I am concerned about the spread of coronavirus (COVID-19) in my community		
Disagree	Ref	Ref
Agree	0.92	0.79–1.07
I am concerned about getting infected with COVID-19		
Disagree	Ref	Ref
Agree	0.99	0.85–1.15

Note: At cohort enrolment, participants were asked if they had used non-pharmaceutical interventions to prevent SARS-CoV-2 in the preceding month. Missing data was addressed using complete-case analysis. Purposeful variable selection was performed to create a model for each outcome and to estimate prevalence ratios and 95% confidence intervals for the association between each independent variable and use of the non-pharmaceutical intervention. Variables in the final model are shown, and statistically significant prevalence ratios (*p* < 0.05) are shown in bold.

SARS-CoV-2, severe acute respiratory syndrome coronavirus 2; Ref, reference; AIC, Akaike information criterion.

**TABLE 3:** Multivariable analyses of factors associated with endorsement of reducing shopping to prevent COVID-19 (*n* = 349, AIC = 694.1).

Variable	Adjusted prevalence ratio	95% confidence interval
**Demographic variables**		
Age (years)		
14–24	Ref	Ref
25–55	**1.13**	**1.00–1.28**
Marital Status		
Not Married	Ref	Ref
Married	**1.19**	**1.01–1.41**
**COVID-19 variables**		
COVID-19 wave		
Non-wave	Ref	Ref
Omicron: 29 November 2021 – 14 February 2022	**0.83**	**0.73–0.96**
Omicron BA.4/BA.5: 02 May 2022 – 22 August 2022	**1.14**	**1.03–1.25**
Omicron BQ.1/BQ.1.1: 10 October 2022 – 02 January 2023	0.95	0.80–1.11
**Knowledge statements**		
I can still have a COVID-19 infection even if I do not have symptoms		
False	Ref	Ref
True	1.10	0.92–1.31
Variants (mutated strains) of COVID-19 may increase the chance that people can be infected with COVID-19		
False	Ref	Ref
True	1.15	0.97–1.37
Variants (mutated strains) of COVID-19 may decrease the effectiveness of our COVID-19 vaccinations		
False	Ref	Ref
True	0.92	0.81–1.05
Wearing a face mask or covering correctly		
Disagree	Ref	Ref
Agree	**0.48**	**0.31–0.76**
Staying at least 1 metre away from other people when out in public		
Disagree	Ref	Ref
Agree	1.04	0.61–1.77
Reducing shopping trips		
Disagree	Ref	Ref
Agree	**1.94**	**1.31–2.87**
Meeting others in open spaces rather than indoors		
Disagree	Ref	Ref
Agree	**1.37**	**1.00–1.87**
**Attitude statements**		
People in my community wear a face mask or covering		
Occasionally or less	Ref	Ref
Always or Often	**1.12**	**1.02–1.23**

Note: At cohort enrolment, participants were asked if they had used non-pharmaceutical interventions to prevent SARS-CoV-2 in the preceding month. Missing was addressed using complete-case analysis. Purposeful variable selection was performed to create a model for each outcome and to estimate prevalence ratios and 95% confidence intervals for the association between each independent variable and use of the non-pharmaceutical intervention. Variables in the final model are shown, and statistically significant prevalence ratios (*p* < 0.05) are shown in bold.

SARS-CoV-2, severe acute respiratory syndrome coronavirus 2; Ref, reference; AIC, Akaike information criterion.

**TABLE 4:** Multivariable analyses of factors associated with endorsement of avoiding crowds to prevent COVID-19 (*n* = 349, AIC = 733.9).

Variable	Adjusted prevalence ratio	95% confidence interval
**Demographic variables**		
Sex		
Female	Ref	Ref
Male	0.99	0.81–1.21
Years of education		
≤ 12	Ref	Ref
> 12	**1.19**	**1.08–1.31**
Men who have sex with men		
No	Ref	Ref
Yes	1.03	0.84–1.26
Engagement in transactional sex		
No	Ref	Ref
Yes	0.93	0.82–1.04
**COVID-19 variables**		
COVID-19 wave		
Non-wave	Ref	Ref
Omicron: 29 November 2021 – 14 February 2022	**0.81**	**0.71–0.93**
Omicron BA.4/BA.5: 02 May 2022–22 August 2022	1.02	0.92–1.12
Omicron BQ.1/BQ.1.1: 10 October 2022 – 02 January 2023	1.00	0.88–1.14
Knows someone with COVID-19		
No	Ref	Ref
Yes	1.04	0.94–1.15
**Knowledge statements**		
COVID-19 can be transmitted through coughing or sneezing		
False	Ref	Ref
True	1.54	0.91–2.62
I can still have a COVID-19 infection even if I do not have symptoms		
False	Ref	Ref
True	1.16	0.96–1.40
People who do not display symptoms may still be infected with COVID-19		
False	Ref	Ref
True	1.01	0.82–1.25
Variants (mutated strains) of COVID-19 may increase the chance that people can be infected with COVID-19		
False	Ref	Ref
True	0.96	0.85–1.09
Washing hands regularly using soap and water or sanitiser (ref: Disagree)		
Disagree	Ref	Ref
Agree	**0.73**	**0.60–0.89**
Avoiding close contact with anyone who has fever and/or cough (ref:)		
Disagree	Ref	Ref
Agree	1.03	0.74–1.42
Staying at least 1 metre away from other people when out in public (ref:)		
Disagree	Ref	Ref
Agree	1.01	0.67–1.54
Reducing shopping trips (ref: Disagree)		
Disagree	Ref	Ref
Agree	0.91	0.73–1.14
Meeting others in open spaces rather than indoors (ref: Disagree)		
Disagree	Ref	Ref
Agree	**1.38**	**1.04–1.84**
Avoiding meetings with crowds including public rallies or religious functions		
Disagree	Ref	Ref
Agree	**1.36**	**1.05–1.77**
**Attitude statements**		
People in my community wear a face mask or covering		
Occasionally or less	Ref	Ref
Always or Often	1.07	0.98–1.17

Note: At cohort enrolment, participants were asked if they had used non-pharmaceutical interventions to prevent SARS-CoV-2 in the preceding month. Missing was addressed using complete-case analysis. Purposeful variable selection was performed to create a model for each outcome and to estimate prevalence ratios and 95% confidence intervals for the association between each independent variable and use of the non-pharmaceutical intervention. Variables in the final model are shown, and statistically significant prevalence ratios (*p* < 0.05) are shown in bold.

SARS-CoV-2, severe acute respiratory syndrome coronavirus 2; Ref, reference; AIC, Akaike information criterion.

For all outcomes, NPI usage varied by COVID-19 wave. Compared to non-wave periods, the initial Omicron-variant wave was associated with less meeting in the open (aPR = 0.85 [95% CI: 0.73–0.98]), reduction in shopping (aPR = 0.83 [95% CI: 0.73–0.96]) and avoidance of crowds (aPR = 0.81 [95% CI: 0.71–0.93]). On the other hand, the second wave was associated with increased probability of reducing shopping (aPR = 1.14 [95% CI: 1.03–1.25]).

Knowledge of the efficacy of personal COVID-19 NPIs was associated with a lower probability of practicing social NPIs. In particular, participants who agreed that wearing face masks or covering correctly prevented the spread of SARS-CoV-2 were less likely to meet in the open or reduce shopping (aPR: 0.44 [95% CI: 0.27–0.73] and 0.48 [95% CI: 0.31–0.76], respectively), and those who agreed that washing hands regularly using soap and water or sanitiser prevents the spread of SARS-CoV-2 were less likely to endorse avoiding crowds (aPR: 0.73 [95% CI: 0.60–0.89]). On the other hand, belief in the efficacy of social NPIs including meeting in the open, reducing shopping and avoiding crowds were each associated with endorsing practicing the same NPI (aPR: 1.57 [95% CI: 1.11–2.21], 1.94 [95% CI: 1.31–2.87], and 1.36 [95% CI: 1.05–1.77], respectively). Finally, participants who believed that people in their communities often wear a face mask or covering were more likely to reduce shopping (aPR: 1.12 [95% CI:1.02–1.23]).

## Discussion

In a cohort of people with behavioural vulnerability to HIV in Kenya, we found that use of NPIs for COVID-19 prevention varied. Generally, the use of personal NPIs including facemasks and handwashing was high, whereas the use of social NPIs including meeting in the open and avoiding crowds was lower. Similarly, although knowledge of COVID-19 NPIs was generally high, knowledge of COVID-19 varied, with higher knowledge of SARS-CoV-2 transmission and lower knowledge of the implications of SARS-CoV-2 genetic variants. In adjusted analyses, whereas knowledge of social NPIs was associated with increased practice of social NPIs, knowledge of personal NPIs was associated with decreased practice of social NPIs. We also found that during the first Omicron SARS-CoV-2 variant wave, participants were less likely to endorse practicing NPIs, but NPI practice increased during the second variant wave. Finally, belief in widespread community facemask use was associated with increased NPI practice.

This study highlights the complex nature of KAPs related to COVID-19 in Kenya, which has been observed in studies from other settings as well.^8,11^ For example, we found evidence of risk compensation wherein individuals who agreed that personal NPIs such as facemasks prevent SARS-CoV-2 transmission were significantly less likely to report reducing shopping or avoiding crowds. This finding aligns with Health Belief Model predictions that individuals balance multiple risk perceptions simultaneously and have a risk threshold that is maintained through behaviours. Therefore, the implementation of an intervention that decreases a health risk (face masks) results in the concomitant increase of another risk behaviour (being in indoor spaces).^34,35,36,37^ Although a global systematic review and meta-analysis of mask-wearing found that none of the studies assessed for risk compensation,^38^ two recent studies from the United States and Bangladesh both found increased amount of time spent outside of the home after mask mandates were put into effect, whereas time spent outside was stable prior to the mask mandate.^36,37^ In the current analyses, many participants engaged in transactional sex, which precludes social distancing, highlighting the economic drivers that simultaneously contribute to COVID-19 and HIV.^39^

This study also demonstrated evidence of community dynamics such as social pressure. Although attitudes regarding personal risk of COVID-19 were not associated with use of NPIs, the perception that other people in the community were using NPIs was associated with increased use of NPIs. This relationship suggests that although individual attitudes tend towards risk compensation, social pressures guide individuals towards uniformity with their communities, which has also been seen in other settings.^40^ In qualitative studies, researchers have suggested that many community-oriented societies in Africa may have decreased social distancing.^41^ Similarly, another study found that survey respondents reported a lack of cultural context for social distancing and physical contact restrictions to hinder engagement in those NPIs.^42^ In the United States, where cultural norms differ from Kenya, a prior study found no evidence of social pressure for mask-wearing, and in fact found that individuals who felt social pressure to wear masks were less likely to wear masks.^43^ The authors of that study attributed the lack of social pressure to multiple possibilities, including fear of violence from masking, fatigue or incomplete knowledge. The influence of community norms on individual behaviour supports social cognitive theory’s emphasis on observational learning and social modelling and further demonstrate the unique influences of culture on the relationships among KAPs.

Finally, we found that the use of social NPIs varied over time, similar to other studies that demonstrated temporal changes in the effectiveness of NPIs.^44^ Early in the pandemic, Kenya had a robust response, enacting restrictions on social gatherings, launching internet-providing balloons to facilitate economy and providing government assistance to businesses to implement sanitation stations.^45^ However, vaccinations were consistently low in Kenya,^46^ so when these measures were relaxed and removed in late 2021, cases of COVID-19 increased.^32,47^ We found that during the initial Omicron-variant wave, individuals were less likely to endorse NPI practice than during non-variant wave periods. This is consistent with a prior study that found a higher infection reproductive number during the first Omicron wave than in the second wave.^48^ However, other than a reduction in shopping during the second Omicron-variant wave, there was no behaviour change in NPIs during subsequent Omicron-variant waves. The patterns of behaviour change during variant waves could reflect changes contributing to increased transmission or changes resulting from increased transmission, which remains to be investigated in future studies.

Because of the overlapping risk factors for HIV and COVID, the current analyses are critical to decreasing morbidity and mortality during future respiratory pandemics. However, there were several limitations to the study. Firstly, practicing NPIs was self-reported and referred to the month prior to survey administration, leading to possible recall bias and misreporting. While misreporting would affect our estimate of the prevalence of each NPI, the associations evaluated in multivariable modelling are unlikely to be affected. Furthermore, we limited our interpretations to the directionality of the adjusted PR rather than the magnitude. Selection bias may also have occurred through clinic-based recruitment, potentially excluding individuals who do not access healthcare services regularly or who avoid clinical settings because of stigma. This could lead to overrepresentation of individuals with better healthcare engagement and potentially different NPI practices. Social desirability bias may also have influenced self-reported NPI practices, with participants potentially over-reporting socially acceptable behaviours such as mask-wearing and handwashing. This bias could inflate prevalence estimates of NPI use and affect associations between knowledge, attitudes and reported practices. Furthermore, eight participants did not complete the COVID-19 questionnaire for unknown reasons, but they represent less than 2% of questionnaires and therefore the potential for selection bias is likely to be small. Secondly, we were limited to cross-sectional variables from a pre-defined questionnaire as part of a larger study that was not focused on COVID-19. The questions did, however, cover a broad swath of knowledge and attitudes to characterise KAPs related to COVID-19. Finally, these analyses used data from a cohort of Kenyan participants with behavioural vulnerabilities to HIV rather than the general population and therefore findings may not be generalisable to the broader Kenyan population, other sub-Saharan African countries with different cultural contexts or HIV-vulnerable populations in different socioeconomic settings.

Although the peak of the COVID-19 pandemic has passed, future respiratory pandemics remain likely. Individual use of NPIs will be critical to controlling disease transmission, and this study demonstrates the complexities of KAPs related to NPI use, including differences between personal NPIs and social NPIs. Furthermore, future public health interventions will need to account for risk-compensating behaviours that decrease NPI use, as well as social pressures that may increase NPI use. For example, when mandating personal NPIs such as facemasks, policymakers should expect and account for likely increases in social risks such as social gatherings by increasing the availability and accessibility of open public spaces. Policies can also focus on messaging of community NPI use to take advantage of social pressures that increase NPI use. However, more research is needed to understand the specific temporal changes during waves of disease transmission given that differentiating between behaviour changes that are causes or effects of waves is difficult in observational studies. Although future pandemics are likely, applying lessons learned from the COVID-19 pandemic, particularly those relating to NPIs and behaviours such as accounting for risk compensation and leveraging social pressures, may mitigate future mortality.^49,50,51^

## Conclusion

We found that in a cohort of individuals with behavioural vulnerabilities to HIV, the practice of NPIs for SARS-CoV-2 prevention was generally high, but cognitive biases such as risk compensation may account for patterns of decreased NPI practice. However, strategies to address risk compensation such as emphasising social pressures may mitigate non-use of NPIs. As individuals with higher risk of HIV also experience higher risk of COVID-19, these results can inform public health interventions in future pandemics.

## Figures and Tables

**FIGURE 1-A1:**
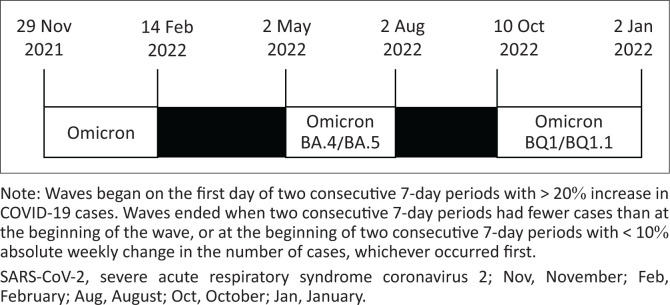
Timeline of COVID-19 waves in Kenya and the corresponding predominant SARS-CoV-2 variant.

## Appendix 1

**TABLE 1-A1:** Knowledge of the effectiveness of non-pharmaceutical interventions (NPIs) for preventing SARS-CoV-2 transmission.

NPI	Strongly agree		Somewhat agree		Neither agree nor disagree		Somewhat disagree		Strongly disagree		Don’t know		Refuse to answer	
	*n*	%	*n*	%	*n*	%	*n*	%	*n*	%	*n*	%	*n*	%
Washing hands	326	82.7	17	4.3	1	0.3	3	0.8	43	10.9	3	0.8	1	0.3
Avoiding contact	327	82.0	17	4.3	1	0.3	7	1.8	40	10.0	7	1.8	0	0.0
Mask-wearing	335	84.6	18	4.5	3	0.8	5	1.3	35	8.8	0	0.0	0	0.0
Social distancing	328	82.8	19	4.8	0	0.0	8	2.0	33	8.3	8	2.0	0	0.0
Meeting in open	304	76.8	22	5.6	4	1.0	11	2.8	45	11.4	10	2.5	0	0.0
Reducing shopping	295	74.3	30	7.6	4	1.0	9	2.3	40	10.1	17	4.3	2	0.5
Avoiding crowds	309	77.8	27	6.8	3	0.8	10	2.5	35	8.8	13	3.3	0	0.0

Note: Participants completed surveys on enrolment that included the following question: ‘For each behaviour, please indicate the extent to which you agree or disagree that it prevents the spread of COVID-19’. Percentages indicate row percent.

SARS-CoV-2, severe acute respiratory syndrome coronavirus 2.

**TABLE 2-A1:** Knowledge of statements describing COVID-19 transmission and genetic variants.

Statement	True		False		Don’t know		Refuse to answer	
	*n*	%	*n*	%	*n*	%	*n*	%
COVID-19 can be transmitted through coughing or sneezing	384	96.7	6	1.5	7	1.8	0	0.0
I can have a COVID-19 infection without having symptoms	333	83.7	33	8.3	32	8.0	0	0.0
People who do not display symptoms may have and spread COVID-19	350	87.9	17	4.3	30	7.5	1	0.3
COVID-19 variants may increase chances of multiple infections	309	77.4	17	4.3	71	17.8	2	0.5
COVID-19 variants may decrease COVID-19 vaccine effectiveness	268	67.5	40	10.1	88	22.2	1	0.3

Note: Participants completed surveys on enrolment that included the following question: ‘Please classify the following statements as either “true” or “false”’. Percentages indicate row percentage.

**TABLE 3-A1:** Attitudes regarding COVID-19 risk.

Statement	Strongly agree		Somewhat agree		Neither agree nor disagree		Somewhat disagree		Strongly disagree		Don’t know		Refuse to answer	
	*n*	%	*n*	%	*n*	%	*n*	%	*n*	%	*n*	%	*n*	%
Becoming infected with COVID-19 poses a risk to others	251	63.1	36	9.0	6	1.5	13	3.3	76	19.1	16	4.0	0	0.0
I am concerned about the spread of COVID-19 in my community	222	55.6	48	12.0	9	2.3	14	3.5	68	17.0	37	9.3	1	0.3
I am concerned about getting infected with COVID-19	217	54.7	46	11.6	9	2.3	12	3.0	56	14.1	55	13.9	2	0.5

Note: Participants completed surveys on enrolment that included the following question: ‘Please select the answer depending on how much you agree with each statement below’. Percentages indicate row percentage.

**TABLE 4-A1:** Belief of frequency of non-pharmaceutical intervention (NPI) practice among members of the community.

NPI	Always		Often		Occasional		Rarely		Never		Don’t know	
	*n*	%	*n*	%	*n*	%	*n*	%	*n*	%	*n*	%
Social distancing	135	33.8	36	9.0	53	13.3	143	35.8	23	5.8	9	2.3
Mask-wearing	145	36.3	43	10.8	60	15.0	127	31.8	18	4.5	6	1.5

Note: Participants completed surveys on enrolment that included the following question: ‘In your community, to what extent do you think that people do the following when they go out in public?’ Percentages indicate row percentage.

NPI, non-pharmaceutical intervention.

**TABLE 5-A1:** Endorsement of practice of non-pharmaceutical interventions to prevent SARS-CoV-2 transmission.

Variable	Yes		No		Don’t know		Refuse to answer	
	*n*	%	*n*	%	*n*	%	*n*	%
Washing hands	390	98.0	5	1.3	3	0.8	0	0.0
Avoiding contact	365	92.4	22	5.6	8	2.0	0	0.0
Mask-wearing	380	95.7	16	4.0	1	0.3	0	0.0
Social distancing	372	94.2	17	4.3	5	1.3	1	0.3
Meeting in open	312	79.2	73	18.5	8	2.0	1	0.3
Reducing shopping	325	81.5	61	15.3	11	2.8	2	0.5
Avoiding crowds	331	84.0	56	14.2	7	1.8	0	0.0

Note: Participants completed surveys on enrolment that included the following question: ‘In the past month, what measures have you taken to prevent infection from COVID-19?’ Percentages indicate row percentage.

SARS-CoV-2, severe acute respiratory syndrome coronavirus 2.

**TABLE 6-A1:** Univariable analyses of meeting in the open on all potential independent variables (*n* = 349).

Variable (Reference)	Unadjusted prevalence ratio	95% confidence interval
**Demographic Variables**		
Age (years)		
14–24	Ref	Ref
25–55	0.97	0.85–1.11
Sex		
Female	Ref	Ref
Male	1.04	0.92–1.17
Site		
Homa Bay	Ref	Ref
Kericho	**1.14**	**1.03–1.27**
Marital status		
Not married	Ref	Ref
Married	1.01	0.76-1.33
Years of education		
≤ 12	Ref	Ref
> 12	1.02	0.92–1.14
Men who have sex with men		
No	Ref	Ref
Yes	1.06	0.91–1.22
Engagement in transactional sex		
No	Ref	Ref
Yes	1.16	0.99–1.37
Weekly income		
≤ 1500.00 Kenyan shilling	Ref	Ref
> 1500.00 Kenyan shilling	0.98	0.88–1.09
**COVID-19-variables**		
COVID-19 wave		
Non-wave	Ref	Ref
Omicron: 29 November 2021 – 14 February 2022	1.04	0.87–1.23
Omicron BA.4/BA.5: 02 May 2022 – 22 August 2022	0.92	0.81–1.04
Omicron BQ.1/BQ.1.1: 10 October 2022 – 02 January 2023	0.96	0.83–1.10
Previously infected with COVID-19		
No	Ref	Ref
Yes	0.96	0.72–1.28
Knows someone with COVID-19 (Yes vs. No)		
No	Ref	Ref
Yes	0.95	0.83–1.10
**Knowledge statements**		
COVID-19 can be transmitted through coughing or sneezing		
False	Ref	Ref
True	1.72	0.95–3.09
I can still have a COVID-19 infection even if I do not have symptoms		
False	Ref	Ref
True	1.15	0.97–1.37
People who do not display symptoms may still be infected with COVID-19		
False	Ref	Ref
True	**1.26**	**1.01–1.57**
Variants (mutated strains) of COVID-19 may increase the chance that people can be infected with COVID-19		
False	Ref	Ref
True	**1.24**	**1.06–1.45**
Variants (mutated strains) of COVID-19 may decrease the effectiveness of our COVID-19 vaccinations		
False	Ref	Ref
True	**1.21**	**1.06–1.38**
Washing hands regularly using soap and water or sanitiser		
Disagree	Ref	Ref
Agree	1.07	0.90–1.27
Avoiding close contact with anyone who has fever and/or cough		
Disagree	Ref	Ref
Agree	1.15	0.96–1.39
Wearing a face mask or covering correctly		
Disagree	Ref	Ref
Agree	0.92	0.80–1.07
Staying at least 1 metre away from other people when out in public		
Disagree	Ref	Ref
Agree	1.23	0.99–1.51
Reducing shopping trips		
Disagree	Ref	Ref
Agree	**1.26**	**1.05–1.52**
Meeting others in open spaces rather than indoors		
Disagree	Ref	Ref
Agree	**1.37**	**1.12–1.66**
Avoiding meetings with crowds including public rallies or religious functions		
Disagree	Ref	Ref
Agree	1.19	0.99–1.43
**Attitude statements**		
My becoming infected with COVID-19 poses a risk to others		
Disagree	Ref	Ref
Agree	1.03	0.91–1.16
I am concerned about the spread of coronavirus (COVID-19) in my community		
Disagree	Ref	Ref
Agree	0.92	0.83–1.02
I am concerned about getting infected with COVID-19		
Disagree	Ref	Ref
Agree	0.93	0.84–1.03
People in my community maintain a distance of at least 1 metre (3 feet)		
Occasionally or less	Ref	Ref
Always or often	0.97	0.87–1.07
People in my community wear a face mask or covering		
Occasionally or less	Ref	Ref
Always or often	0.96	0.87–1.07

Note: At cohort enrolment, participants were asked if they had used each of the non-pharmaceutical interventions to prevent SARS-CoV-2 in the preceding month. Robust Poisson regression was used to estimate prevalence ratios and 95% confidence intervals for the association between each independent variable and use of the non-pharmaceutical intervention. Statistically significant prevalence ratios (*p* < 0.05) are shown in bold.

SARS-CoV-2, severe acute respiratory syndrome coronavirus 2; Ref, reference.

**TABLE 7-A1:** Univariable analyses of reducing shopping on all potential independent variables (*n* = 349).

Variable (Reference)	Unadjusted prevalence ratio	95% confidence interval
**Demographic variables**		
Age (years)		
14–24	Ref	Ref
25–55	1.10	0.99–1.21
Sex		
Female	Ref	Ref
Male	1.06	0.96–1.18
Site		
Homa Bay	Ref	Ref
Kericho	0.98	0.89–1.08
Marital status		
Not Married	Ref	Ref
Married	1.14	0.98–1.33
Years of education		
≤ 12	Ref	Ref
> 12	0.97	0.88–1.08
Men who have sex with men		
No	Ref	Ref
Yes	1.01	0.88–1.17
Engagement in transactional sex		
No	Ref	Ref
Yes	0.95	0.85–1.06
Weekly income		
≤ 1500.00 Kenyan shilling	Ref	Ref
> 1500.00 Kenyan shilling	1.00	0.91–1.10
**COVID-19-variables**		
COVID-19 wave		
Non-wave	Ref	Ref
Omicron: 29 November 2021 – 14 February 2022	0.97	0.82–1.15
Omicron BA.4/BA.5: 02 May 2022 – 22 August 2022	**0.85**	**0.76–0.96**
Omicron BQ.1/BQ.1.1: 10 October 2022 – 02 January 2023	0.98	0.88–1.10
Previously infected with COVID-19		
No	Ref	Ref
Yes	0.92	0.69–1.22
Knows someone with COVID-19 (Yes vs. No)		
No	Ref	Ref
Yes	0.98	0.87–1.11
**Knowledge statements**		
COVID-19 can be transmitted through coughing or sneezing		
False	Ref	Ref
True	1.34	0.87–2.06
I can still have a COVID-19 infection even if I do not have symptoms		
False	Ref	Ref
True	**1.28**	**1.07–1.53**
People who do not display symptoms may still be infected with COVID-19		
False	Ref	Ref
True	**1.23**	**1.00–1.50**
Variants (mutated strains) of COVID-19 may increase the chance that people can be infected with COVID-19		
False	Ref	Ref
True	1.20	1.04–1.38
Variants (mutated strains) of COVID-19 may decrease the effectiveness of our COVID-19 vaccinations		
False	Ref	Ref
True	1.11	1.00–1.24
Washing hands regularly using soap and water or sanitiser		
Disagree	Ref	Ref
Agree	1.00	0.87–1.14
Avoiding close contact with anyone who has fever and/or cough		
Disagree	Ref	Ref
Agree	1.17	0.98–1.40
Wearing a face mask or covering correctly		
Disagree	Ref	Ref
Agree	0.91	0.81–1.03
Staying at least 1 metre away from other people when out in public		
Disagree	Ref	Ref
Agree	**1.20**	**0.99–1.45**
Reducing shopping trips		
Disagree	Ref	Ref
Agree	**1.57**	**1.27–1.94**
Meeting others in open spaces rather than indoors		
Disagree	Ref	Ref
Agree	**1.36**	**1.13–1.64**
Avoiding meetings with crowds including public rallies or religious functions		
Disagree	Ref	Ref
Agree	**1.25**	**1.04–1.50**
**Attitude statements**		
My becoming infected with COVID-19 poses a risk to others		
Disagree	Ref	Ref
Agree	0.96	0.87–1.06
I am concerned about the spread of coronavirus (COVID-19) in my community		
Disagree	Ref	Ref
Agree	0.94	0.86–1.03
I am concerned about getting infected with COVID-19		
Disagree	Ref	Ref
Agree	0.94	0.86–1.03
People in my community maintain a distance of at least 1 metre (3 feet)		
Occasionally or less	Ref	Ref
Always or often	1.05	0.95–1.15
People in my community wear a face mask or covering		
Occasionally or less	Ref	Ref
Always or often	1.04	0.94–1.14

Note: At cohort enrolment, participants were asked if they had used each of the non-pharmaceutical interventions to prevent SARS-CoV-2 in the preceding month. Robust Poisson regression was used to estimate prevalence ratios and 95% confidence intervals for the association between each independent variable and use of the non-pharmaceutical intervention. Statistically significant prevalence ratios (*p* < 0.05) are shown in bold.

SARS-CoV-2, severe acute respiratory syndrome coronavirus 2; Ref, reference.

**TABLE 8-A1:** Univariable analyses of avoiding crowds on all potential independent variables (*n* = 349).

Variable (Reference)	Unadjusted prevalence ratio	95% confidence interval
**Demographic variables**		
Age (years)		
14–24	Ref	Ref
25–55	1.02	0.91–1.13
Sex		
Female	Ref	Ref
Male	1.06	0.96–1.17
Site		
Homa Bay	Ref	Ref
Kericho	0.97	0.89–1.07
Marital status		
Not married	Ref	Ref
Married	0.94	0.72–1.25
Years of education		
≤ 12	Ref	Ref
> 12	**1.13**	**1.04–1.23**
Men who have sex with men		
No	Ref	Ref
Yes	1.08	0.96–1.21
Engagement in transactional sex		
No	Ref	Ref
Yes	0.92	0.83–1.01
Weekly income		
≤ 1500.00 Kenyan shilling	Ref	Ref
> 1500.00 Kenyan shilling	0.97	0.89–1.06
**COVID-19-variables**		
COVID-19 wave		
Non-wave	Ref	Ref
Omicron: 29 November 2021 – 14 February 2022	1.07	0.96–1.19
Omicron BA.4/BA.5: 02 May 2022 – 22 August 2022	**0.87**	**0.77–0.97**
Omicron BQ.1/BQ.1.1: 10 October 2022 – 02 January 2023	0.94	0.84–1.05
Previously infected with COVID-19		
No	Ref	Ref
Yes	0.90	0.68–1.20
Knows someone with COVID-19 (Yes vs. No)		
No	Ref	Ref
Yes	1.10	1.00–1.20
**Knowledge statements**		
COVID-19 can be transmitted through coughing or sneezing		
False	Ref	Ref
True	**1.82**	**1.01–3.28**
I can still have a COVID-19 infection even if I do not have symptoms		
False	Ref	Ref
True	**1.24**	**1.05–1.46**
People who do not display symptoms may still be infected with COVID-19		
False	Ref	Ref
True	1.21	1.00–1.46
Variants (mutated strains) of COVID-19 may increase the chance that people can be infected with COVID-19		
False	Ref	Ref
True	**1.18**	**1.03–1.35**
Variants (mutated strains) of COVID-19 may decrease the effectiveness of our COVID-19 vaccinations		
False	Ref	Ref
True	1.08	0.97–1.19
Washing hands regularly using soap and water or sanitiser		
Disagree	Ref	Ref
Agree	0.96	0.85–1.08
Avoiding close contact with anyone who has fever and/or cough		
Disagree	Ref	Ref
Agree	1.16	0.98–1.38
Wearing a face mask or covering correctly		
Disagree	Ref	Ref
Agree	0.96	0.84–1.09
Staying at least 1 metre away from other people when out in public		
Disagree	Ref	Ref
Agree	**1.23**	**1.01–1.48**
Reducing shopping trips		
Disagree	Ref	Ref
Agree	**1.20**	**1.03–1.40**
Meeting others in open spaces rather than indoors		
Disagree	Ref	Ref
Agree	**1.28**	**1.08–1.52**
Avoiding meetings with crowds including public rallies or religious functions		
Disagree	Ref	Ref
Agree	**1.31**	**1.09–1.58**
**Attitude statements**		
My becoming infected with COVID-19 poses a risk to others		
Disagree	Ref	Ref
Agree	1.02	0.92–1.12
I am concerned about the spread of coronavirus (COVID-19) in my community		
Disagree	Ref	Ref
Agree	0.98	0.89–1.07
I am concerned about getting infected with COVID-19		
Disagree	Ref	Ref
Agree	1.00	0.91–1.10
People in my community maintain a distance of at least 1 metre (3 feet)		
Occasionally or less	Ref	Ref
Always or often	1.07	0.98–1.16
People in my community wear a face mask or covering		
Occasionally or less	Ref	Ref
Always or often	1.06	0.97–1.16

Note: At cohort enrolment, participants were asked if they had used each of the non-pharmaceutical interventions to prevent SARS-CoV-2 in the preceding month. Robust Poisson regression was used to estimate prevalence ratios and 95% confidence intervals for the association between each independent variable and use of the non-pharmaceutical intervention. Statistically significant prevalence ratios (*p* < 0.05) are shown in bold.

SARS-CoV-2, severe acute respiratory syndrome coronavirus 2; Ref, reference.
